# Impact of Baseline Kidney Function on the Rate of Progressive Kidney Disease After Pregnancy: A Population-Based Cohort Study Research Protocol

**DOI:** 10.1177/20543581251318836

**Published:** 2025-02-28

**Authors:** Lavanya Bathini, Nivethika Jeyakumar, Jessica Sontrop, Eric McArthur, Yuguang Kang, Bin Luo, Aminu Bello, David Collister, Sofia Ahmed, Padma Kaul, Erik Youngson, Branko Braam, Nir Melamed, Michelle Hladunewich, Amit X. Garg

**Affiliations:** 1 Department of Medicine, University of Alberta, Edmonton, Canada; 2 University of Alberta Hospital, Edmonton, Canada; 3 London Health Sciences Research, ON, Canada; 4 Department of Epidemiology and Biostatistics, Schulich School of Medicine and Dentistry, Western University, London, ON, Canada; 5 ICES, Toronto, ON, Canada; 6 Provincial Research Data Services, Alberta Health Services, Edmonton, Canada; 7 Data and Research Services, Alberta SPOR SUPPORT Unit, Edmonton, Canada; 8 Divisions of Nephrology and Obstetric Medicine, Department of Medicine, Sunnybrook Health Sciences Centre, Temerty School of Medicine, University of Toronto, ON, Canada; 9 Division of Nephrology, Department of Medicine, Schulich School of Medicine and Dentistry, Western University, London, ON, Canada

**Keywords:** pregnancy, kidney outcomes, maternal health, kidney progression

## Abstract

**Background:**

Better data are necessary to determine whether baseline level of kidney function affects the rate of progressive kidney disease following pregnancy.

**Objective:**

The objective was to determine whether the baseline (pre-pregnancy) estimated glomerular filtration rate (eGFR) modifies the association between becoming pregnant and the subsequent rate of progressive kidney disease.

**Design:**

Population-based cohort study using provincial administrative health care databases in Ontario and Alberta, Canada.

**Setting:**

The sample will be accrued from April 1, 2007, to March 31, 2023, in Ontario and from April 1, 2012, to March 31, 2023, in Alberta. Follow-up for study outcomes will occur until March 31, 2024.

**Participants:**

The pregnant group will include adult female residents of Ontario or Alberta with a record of a pregnancy of 20 to 46 weeks’ gestation during the accrual period, and the non-pregnant group will include adult female residents with no prior record of pregnancy. The cohort entry dates in those in the pregnant group will be the estimated date of conception; the entry dates for those in the non-pregnant group will be randomly assigned following the distribution of dates in the pregnant group. To be eligible, individuals must be between 18 and 45 years old at cohort entry. They require at least 1 serum creatinine measurement within 2 years before entry and should not have received maintenance dialysis or a prior kidney transplant. Both groups will be categorized into one of 3 levels of baseline eGFR (≥60, 45–59, and <45 mL/min per 1.73 m^2^). Inverse probability of treatment weighting on a propensity score will be used to balance the pregnant and non-pregnant groups on baseline characteristics (including age, proteinuria, hypertension, and diabetes) within the 3 categories of baseline eGFR.

**Measurements:**

The primary outcome, progressive kidney disease, will be defined as a composite of a persistent ≥40% drop in eGFR from the baseline value, a new persistent eGFR <15 mL/min per 1.73 m^2^, receipt of maintenance dialysis, or receipt of a kidney transplant. The secondary outcomes will be the components of the primary composite outcome examined separately and the annualized change in eGFR in mL/min per 1.73 m^2^ from baseline.

**Methods:**

We will test for statistical interaction to determine whether the baseline category of eGFR modifies the rate of long-term progressive kidney disease after pregnancy. We hypothesize that a statistical interaction will be present. We will present weighted cause-specific hazard ratios (HRs) and cumulative incidence function (CIF) curves for up to 10 years of follow-up for the pregnant and non-pregnant groups stratified by each eGFR category. We will perform additional pre-specified analyses to confirm whether the findings are robust and examine associations that account for baseline proteinuria.

**Results:**

Based on a feasibility analysis using ICES data in Ontario, we expect the cohort to include over 400 000 pregnant females and 1.2 million non-pregnant females. This includes at least 395 000 pregnant females with baseline eGFR ≥60 mL/min/1.73 m^2^, 300 with eGFR 45 to 59 mL/min/1.73 m^2^, and 110 with eGFR <45 mL/min/1.73 m^2^. The median follow-up is anticipated to be 5 years (range = 1-17 years) with minimal loss to follow-up.

**Limitations:**

Measures of kidney function will be obtained as part of routine care (not according to a research schedule). Measures of baseline proteinuria are frequently missing from routine care data, even in up to 15% of those with an eGFR <45 mL/min per 1.73 m^2^.

**Conclusion:**

This study will investigate whether the level of baseline eGFR modifies the rate of progressive kidney disease after pregnancy and will estimate the cumulative incidence of progressive kidney disease in pregnant and non-pregnant females across 3 categories of baseline eGFR.

## Résumé

**Contexte:**

De meilleures données sont nécessaires pour déterminer si le niveau initial de fonction rénale affecte le taux d’insuffisance rénale progressive après la grossesse.

**Objectif:**

Déterminer si le débit de filtration glomérulaire estimé initial (DFGe), soit avant la grossesse, modifie l’association entre la grossesse et le taux subséquent (après la grossesse) d’insuffisance rénale progressive.

**Type d’étude:**

Étude de cohorte représentative d’une population réalisée à partir des données administratives de santé provinciales de l’Ontario et de l’Alberta (Canada).

**Cadre:**

Les sujets des échantillons seront comptabilisés entre le 1^er^ avril 2007 et le 31 mars 2023 en Ontario et entre le 1^er^ avril 2012 et le 31 mars 2023 en Alberta. Les résultats de l’étude seront suivis jusqu’au 31 mars 2024.

**Personnes participantes:**

Le groupe des femmes enceintes regroupera des résidentes de l’Ontario ou de l’Alberta dont le dossier indique une grossesse de 20 à 46 semaines pendant la période de cumul de données. Le groupe des femmes non enceintes regroupera des sujets aux caractéristiques similaires sans grossesse antérieure au dossier. Pour les femmes enceintes, la date d’entrée dans la cohorte sera la date estimée de la conception; les femmes non enceintes se verront attribuer aléatoirement une date d’entrée dans la cohorte en fonction de la répartition des dates dans le groupe des femmes enceintes. Pour être admissibles, les femmes doivent être âgées de 18 à 45 ans au moment de l’entrée dans la cohorte, avoir au moins une mesure de la créatininémie dans les deux ans précédant leur inclusion et ne pas avoir reçu de dialyse d’entretien ou avoir subi de greffe de rein antérieurement. Les deux groupes seront classés selon trois niveaux de DFGe initial (≥60, 45 à 59 et <45 ml/min par 1,73 m²). La pondération de la probabilité inverse du traitement sur le score de propension sera utilisée pour équilibrer les groupes de femmes enceintes et non enceintes selon leurs caractéristiques initiales (p. ex., âge, protéinurie, hypertension, diabète) pour les trois niveaux de DFGe initial.

**Mesures:**

Le principal critère d’évaluation, l’insuffisance rénale progressive, a été défini comme un composite des éléments suivants : réduction persistante d’au moins 40 % du DFGe par rapport à la valeur initiale, DFGe nouvellement établi à < 15 ml/min/1,73 m² de façon persistante, dialyse d’entretien ou transplantation rénale. Les critères d’évaluation secondaires seront les composantes de ce critère composite examinées séparément, de même que la variation annualisée du DFGe (ml/min par 1,73 m²) par rapport à la valeur initiale.

**Méthodologie:**

Nous testerons l’interaction statistique afin de déterminer si le niveau de DFGe initial modifie le taux d’insuffisance rénale progressive à long terme après la grossesse. Nous émettons l’hypothèse qu’une interaction statistique sera présente. Nous présenterons les risques relatifs (RR) pondérés par cause et les courbes de la fonction d’incidence cumulative (FIC) pour une période de suivi allant jusqu’à 10 ans pour les deux groupes, stratifiés par chaque catégorie de DFGe initial. Nous procéderons à des analyses supplémentaires préspécifiées afin de confirmer la robustesse des résultats et nous examinerons les associations pouvant expliquer la protéinurie initiale.

**Résultats:**

Une analyse de faisabilité réalisée à l’aide des données du répertoire de l’ICES en Ontario nous permet d’estimer que la cohorte comprendra plus de 400 000 femmes enceintes et 1,2 million de femmes non enceintes. La cohorte des femmes enceintes comprendra au moins 395 000 femmes avec un DFGe initial ≥60 ml/min/1,73 m², 300 avec un DFGe de 45 à 59 ml/min/1,73 m² et 110 avec un DFGe < 45 ml/min/1,73 m². Le suivi médian devrait être de 5 ans (intervalle : 1 à 17 ans) avec une perte minimale de suivi.

**Limites:**

Les mesures de la fonction rénale seront obtenues dans le cadre des soins courants (et non selon un calendrier de recherche). Les mesures initiales de protéinurie sont souvent absentes des données des soins courants, et ce, même pour jusqu’à 15 % des personnes présentant un DFGe < 45 ml/min/1,73 m².

**Conclusion:**

Cette étude vise à déterminer si le DFGe initial modifie le taux d’insuffisance rénale progressive après la grossesse. Elle évaluera également l’incidence cumulative d’insuffisance rénale progressive chez les femmes enceintes et non enceintes pour trois catégories de DFGe initial.

## Background

Women with chronic kidney disease (CKD) face emotional distress, as they weigh their desire for a child against the need to preserve kidney function.^1^ The long-term impact of pregnancy on CKD progression is unclear, although data suggest pregnancy may hasten the time to kidney failure.^1-4^ This may be due to glomerular hyperfiltration, disease relapse, discontinuation of teratogenic kidney medications, and complications like pre-eclampsia.^2-6^

Although pregnant individuals with mild CKD likely have a comparable kidney prognosis to those without CKD, prior retrospective studies suggest that 1 in 3 women with advanced CKD may lose 25% or more of their eGFR or require dialysis within 6 months of delivery.^2,3^ The largest study to date demonstrated that pregnancy accelerates the need for dialysis or transplantation by 2.5 to 5 years in women with advanced CKD.^4^ We lack the necessary data to counsel persons with CKD on long-term kidney disease risk after pregnancy, as all existing studies lack comparisons with non-pregnant individuals with similar baseline kidney function.^1-4^

We will conduct a retrospective cohort study using health care data from Ontario and Alberta, including at least 400 000 pregnant females (≥20 weeks gestation) and 1.2 million non-pregnant females. Statistical weighting will balance baseline health indicators. The median follow-up time is expected to be 5 years (maximum 17 years).

## Objectives

### Primary Objective

1. To determine whether the category of baseline (pre-pregnancy) eGFR modifies the association between becoming pregnant and the rate of long-term progressive kidney decline. Baseline eGFR will be categorized as ≥60, 45–59, and <45 mL/min per 1.73 m^2^.

We hypothesize there will be statistical interaction based on the relative excess risk due to interaction (RERI) estimated using hazard ratios (HRs). We will estimate weighted cause-specific HRs and cumulative incidence functions (CIFs) for the pregnant vs non-pregnant groups separately for each of the 3 categories of baseline eGFR. We expect the absolute difference in cumulative incidence between the pregnant and non-pregnant groups to be greatest in the lowest category of eGFR (eGFR <45 mL/min per 1.73 m^2^) compared with the other 2 categories of eGFR.

### Secondary Objectives

2. To determine whether the category of baseline (pre-pregnancy) eGFR modifies the association between becoming pregnant and the annualized change in eGFR in mL/min per 1.73 m^2^.

We will estimate the annualized change in eGFR in pregnant vs non-pregnant females separately for each of the 3 categories of baseline eGFR. We hypothesize statistical interaction, with the greatest decline expected in the lowest eGFR category (<45 mL/min/1.73 m^2^) compared with higher categories.

3. To determine whether the category of baseline proteinuria, beyond the category of baseline eGFR, further modifies the association between becoming pregnant and the rate of progressive kidney disease.

We hypothesize there will be statistical interaction. We will estimate weighted cause-specific HRs and cumulative incidence functions (CIFs) in the pregnant vs non-pregnant groups for each eGFR and proteinuria category. We expect within each eGFR category (and particularly in the lowest categories) that the absolute difference in cumulative incidence will be the greatest in the highest category of proteinuria.

## Methods

### Design and Setting

We will conduct a population-based cohort study using linked health care data from Ontario and Alberta, which provide universal health care. In 2021, the combined population of these provinces was 18.5 million, with 17% of them being females aged 20 to 44 years.^5^ The sample will be accrued from April 1, 2007, to March 31, 2023, in Ontario, and April 1, 2012, to March 31, 2023, in Alberta, reflecting differences in lab data availability. Outcomes will be followed until March 31, 2024, with follow-up ranging from 1 to 17 years.

To comply with privacy regulations for minimizing the chance of patient identification, specific values of cells with 5 or fewer individuals will be suppressed (reported as <6). The study will be conducted and reported following recommended guidelines for observational studies using routinely collected health data (eTable 1 in Supplemental Appendix).^6,7^

Owing to data restrictions, person-level data sets cannot be combined across provinces. Analyses will be conducted separately in each province, with results aggregated using described techniques. Any inter-provincial heterogeneity in results is expected to arise from baseline population differences.

### Participants

The study will consist of 2 groups. The pregnant group will consist of female Ontario or Alberta residents with a recorded pregnancy of 20 to 46 weeks’ gestation during the accrual period. The non-pregnant group will consist of female residents with no pregnancy record in our data sets which includes the accrual and follow-up periods. For the pregnant group, the *cohort entry date* (index date 1) will be the estimated date of conception. Index date 2 will be the date of delivery. For the non-pregnant group, the cohort entry date will be randomly assigned based on the distribution of days between the most recent serum creatinine record and the pregnant group’s conception date. Similarly, a fake delivery date (index date 2) will be randomly assigned in the non-pregnant group based on the distribution of days between the conception and delivery dates in the pregnant group (eFigure 1 in Supplemental Appendix).

#### Selection criteria

To enter, all individuals must have at least 1 serum creatinine measurement in the 2 years prior to cohort entry. We will then apply the exclusion criteria listed below. Preliminary numbers from the feasibility analysis in Ontario are shown in eFigure 1 (Supplemental Appendix).

For the pregnant group, exclusions will be applied to records in the MOMBABY and Alberta Perinatal Health Program data sets.^8,9^ To ensure clear clinical interpretation, only the most recent eligible pregnancy per female will be included. Other methods, such as treating pregnancies as time-dependent factors, were considered but deemed overly complex. For the non-pregnant group, study selection criteria unrelated to pregnancy will be applied, and a single record per individual will be randomly selected.

#### Exclusion criteria

(1) Data cleaning: Records with errors (eg, erroneous age, missing sex, maternal death or non-provincial resident before cohort entry, births <20 or >46 weeks’ gestation [to ensure all deliveries captured in the mother-baby linked data sets are represented in our study], and poor data quality concerning the delivery [ie, invalid or discrepant baby unique identifiers, and record of another birth within 3 and 140 days of the index delivery date as it would be impossible to carry 2 viable pregnancies during this time]). We anticipate that less than 1% of the records will be excluded due to data cleaning. (2) Age <18 or ≥45 (as pregnancies in individuals >45 with an eGFR < 45 mL/min per 1.73 m^2^ are rare). (3) Prior hysterectomy or congenital absence of the uterus (conditions rendering pregnancy impossible). (4) Unstable baseline eGFR: inpatient serum creatinine tests differing by >10 mL/min/1.73 m^2^ from outpatient tests. We have previously demonstrated that outpatient serum creatinine measurements in the province of Ontario, conducted on a single occasion, indicate stable values.^10^ (5) History of kidney failure (receipt of maintenance dialysis of ≥3 months or a kidney transplant). (6) Evidence of another pregnancy in follow-up (the primary analysis will be restricted to the last eligible pregnancy for ease of clinical interpretation).

For the non-pregnant group, we will also exclude those whose baseline eGFR value is lower than the lowest recorded value in the pregnant group (expected to be <0.1% of the records of the non-pregnant group).

#### Statistical weighting

We will use inverse probability of treatment weighting on the propensity score to balance baseline characteristics between pregnant and non-pregnant groups, enabling analysis of the full data set. Preliminary density plots for eGFR categories (≥60, 45–59, <45 mL/min/1.73 m^2^) showed good overlap after weighting.

The propensity score is the probability of being in the pregnant group versus the non-pregnant group, conditional on several baseline characteristics. We will use propensity scores to weight females in the non-pregnant group using average treatment effect for the treated (ATT) weights (where treatment/treated refers to pregnant), defined as [propensity score/(1 − propensity score)].^11-14^ Those in the pregnant group will receive weights of 1. Separate propensity score models will be used for each eGFR category to account for baseline health differences, such as higher diabetes and hypertension prevalence in those with eGFR <45 mL/min/1.73 m^2^.

Propensity scores will be calculated using multivariable logistic regression, including baseline factors such as age, cohort entry year, eGFR, history of hypertension and diabetes, and proteinuria category (missing, normal/mild, moderate, severe; see eTable 2 in Supplemental Appendix).^15^ Variable inclusion will be based on known associations with pregnancy and kidney disease. If the model fails to balance baseline characteristics, adjustments will be made before outcome analysis. Balancing advanced age, diabetes, hypertension, and severe proteinuria is crucial, as these factors increase the risk of kidney function decline.

#### Exposure

This study’s exposure is a pregnancy between 20 and 46 weeks of gestation. The non-pregnant group will be the reference group in all analyses.

#### Outcomes

The primary outcome is progressive kidney disease, defined as a composite of a persistent ≥40% drop in eGFR from the baseline value, a new persistent eGFR <15 mL/min per 1.73 m^2^, or receipt of kidney replacement therapy (ie, receipt of maintenance dialysis or a kidney transplant). Persistence refers to evidence of at least 2 eGFR measurements separated by at least 90 days but no more than 365 days. The secondary outcomes will be the components of the primary composite outcome examined separately and the annualized change in eGFR in mL/min per 1.73 m^2^/year from baseline. Outcomes will be assessed starting from the cohort entry date (date of conception/simulated date of conception). Females will be followed for study outcomes until loss to follow-up, death, or the end of the study period, whichever comes first. Emigration from the province is the only reason for lost follow-up. We anticipate this rate to be less than 1.5% per year.

#### Justification for the choice of primary and secondary outcomes

The primary composite outcome includes kidney failure (a clinically significant endpoint) and a ≥40% decline in eGFR (a validated surrogate for kidney failure) to ensure adequate statistical power, as detecting differences in kidney failure alone would require a much larger sample.^16,17^

Previous studies, limited by small sample sizes (median pregnancies = 130), focused on eGFR changes and CKD stage progression.^1-4^ To enable comparisons, we will also assess annualized eGFR change, considering a difference of ≥0.75 mL/min/1.73 m^2^/year clinically meaningful, as prior studies have established this level is a valid surrogate endpoint for CKD progression.^18,19^

### Data Sources, Linkage, and Study Variables

All data for this study will come from linked provincial administrative health care databases in Ontario and Alberta. A description of the databases is provided in Table 1. Within each province, individual-level data will be linked across databases using unique encoded identifiers. Access to Alberta data will be coordinated through the Data Access Support Hub (DASH), available through the Health Disease Research Network (HDRN). Details on the administrative data codes that will be used to define baseline characteristics and other study variables are provided in eTable 2 (Supplemental Appendix).

**Table 1.** Description of Study Databases.

Database	Description
National Databases/Registries	
Canadian Institute for Health Information’s Discharge Abstract Database/ Same Day Surgery (CIHI-DAD/SDS)	Contains diagnostic and procedural information for all hospitalizations; collected by each province.
Canadian Organ Replacement Registry (CORR)	National registry that includes information on vital organ transplantation and dialysis activities.
National Ambulatory Care Reporting System (NACRS)	Contains information on hospital and community-based ambulatory care visits.
Ontario Databases	
Ontario Diabetes Dataset (ODD)	ICES derived data set that includes all patients with diabetes in Ontario.
Ontario Mental Health Reporting System (OMHRS)	Database contains adult inpatient mental health information.
Registered Persons Database (RPDB)	Contains information on patient demographics including sex, birth, and death dates.
ICES Physician Database (IPDB)	Database contains physician-related information such as birth date, sex, education, and specializations.
Ontario Hypertension Dataset (HYPER)	ICES derived data set that includes all patients with hypertension in Ontario.
Ontario Mother-Baby Linked Dataset (MOMBABY)	Contains linked inpatient admission records of delivering mothers and their newborns.
Ontario Health Insurance Plan (OHIP)	Contains diagnostic information and health claims for inpatient and outpatient services.
Ontario Laboratories Information System (OLIS)	Contains laboratory test orders and results from hospitals, community labs, and public health labs.
Alberta Databases	
Alberta Health Care Insurance Plan Registry (AHCIP)	Year of birth, gender, and postal code for all Alberta residents registered for provincial health insurance.
Alberta Health Practitioner Claims	Diagnostic information and health claims for inpatient and outpatient services.
Alberta Perinatal Health Program (APHP)	Maternal, obstetrical, and neonatal information during the antepartum, intrapartum, and postpartum periods for all deliveries.
Provincial Laboratory Data (PLD)	All general lab tests (both inpatient and outpatient). Includes results for most clinical chemistry, toxicology, hematology, serology, urinalysis, and immunology tests.
Diagnostic Imaging (DI)	Records of diagnostic imaging conducted at Alberta Health Services facilities.
Vital Statistics	Contains dates and causes of death.

*Note.* CIHI-DAD/SDS = Canadian Institute for Health Information’s Discharge Abstract Database/ Same Day Surgery; CORR = Canadian Organ Replacement Registry; NACRS = National Ambulatory Care Reporting System; ODD = Ontario Diabetes Dataset; OMHRS = Ontario Mental Health Reporting System; RPDB = Registered Persons Database; IPDB = ICES Physician Database; HYPER = Ontario Hypertension Dataset; MOMBABY = Ontario Mother-Baby linked Dataset; OHIP = Ontario Health Insurance Plan; OLIS = Ontario Laboratories Information System; AHCIP = Alberta Health Care Insurance Plan Registry; APHP = Alberta Perinatal Health Program; PLD = Provincial Laboratory Data; DI = Diagnostic Imaging.

We will obtain patient characteristics and laboratory data from multiple linked health care databases in Ontario (ICES) and Alberta. Information on livebirths and stillbirths in Ontario is available in the mother-baby linked data set (MOMBABY) and the Alberta Perinatal Health Program database (APHP). The Ontario Laboratory Information System (OLIS) and Alberta’s Provincial Laboratory Data (PLD) database provide information on serum creatinine values. Ontario’s Registered Persons Database (RPDB) and the Alberta Health Care Insurance Population Registry (AHCIP) provide patient characteristics and vital statistics information. Information on hospital admissions and diagnoses is present in the Canadian Institute for Health Information Discharge Abstract Database (CIHI-DAD), the Ontario Health Insurance Plan (OHIP) database, and the Alberta Practitioner Claims database. These databases have been used extensively for epidemiologic and health services research, including maternal and fetal outcome studies.^20,21^

Baseline characteristics will be assessed at cohort entry and include age, rural vs urban residence, neighborhood income quintile, comorbidities recorded in the past 5 years (eg, diabetes mellitus, hypertension, the Charlson Comorbidity Index), and general health care use in the year before cohort entry (eg, number of family physician and nephrologist visits). Comorbidities will be defined using the International Statistical Classification of Diseases and Related Health Problems, 10th revision (ICD-10-CA), and diagnostic and fee codes from physician claims databases (eTable 2 in Supplemental Appendix). Baseline eGFR will be calculated from the most recent serum creatinine test within 2 years before cohort entry using the 2021 Chronic Kidney Disease Epidemiology (CKD-EPI) equation (recalibrated to remove an indicator for race).^22^ All serum creatinine testing in the provinces was traceable to isotope dilution mass spectrometry (IDMS) during the study period. The most recent proteinuria test result in the 2 years before the cohort entry date will be categorized using a hierarchical combination of the albumin-to-creatinine ratio (ACR), protein-to-creatinine ratio (PCR), and dipstick proteinuria based on data availability (ie, if an ACR measurement is available, this measurement will be used over a PCR or urine dipstick measurement). Proteinuria will be categorized as missing, normal/mild, moderate, or severe, as defined in Table 2.^23^ We expect a measurement of proteinuria when assessed this way to be present in approximately 50% of those with an eGFR ≥60 mL/min per 1.73 m^2^ and 75% and 85% of those with an eGFR between 45 and 59 and <45 mL/min per 1.73 m^2^, respectively.^24^

**Table 2.** Proteinuria Categories and Definition.

Category	Definition
Normal/mild	Undetectable ACR/PCR values, ACR value <3 mg/mmol, PCR value <5 mg/mmol, or urine dipstick negative/trace
Moderate	ACR value 3-30 mg/mmol, PCR value 15-50 mg/mmol, or urine dipstick 1+
Severe	ACR >30 mg/mmol, PCR >50 mg/mmol, or urine dipstick ≥2+
Missing	No test result

*Note.* ACR = albumin-to-creatinine ratio; PCR = protein-to-creatinine ratio.

In the pregnant group, we will describe pregnancy-specific characteristics present on the date of delivery, including pre-eclampsia/eclampsia, gestational hypertension, gestational diabetes, parity, and multiple gestations. Pregnancy-related health care use will also be described from conception (cohort entry) to delivery.

For individuals with drug coverage through provincial health care plans, we will describe the percentage in each eGFR category receiving medications that block the renin-angiotensin-aldosterone system. This will be described both at baseline and during the follow-up period (for the latter, see the additional analyses section below). In Ontario, we expect approximately 15% of females with an eGFR ≥ 60 mL/min per 1.73 m^2^ will have such coverage at cohort entry, and 25% and 40% of females with an eGFR between 45 and 59 and <45 mL/min per 1.73 m^2^ will have such coverage, respectively. In Ontario, among those with coverage, we expect less than 1% of females with an eGFR ≥ 60 mL/min per 1.73 m^2^ will have evidence of baseline use of drugs which block the renin-angiotensin-aldosterone system. Corresponding values for females with a baseline eGFR between 45 and 59 and <45 mL/min per 1.73 m^2^ are expected to be 13% and 21%, respectively. The use of immunosuppression, sodium-glucose cotransporter-2 (SGLT-2) inhibitors, non–steroidal mineralocorticoid receptor antagonists (nsMRAs), and glucagon-like peptide-1 (GLP-1) receptor agonists is expected to be too limited for inclusion due to the study’s timeframe and restricted drug data availability.

As our primary outcome relies on serum creatinine measurements, we will report the rate of serum creatinine testing in follow-up as done in routine care by group in each eGFR category.

### Statistical Analysis Plan

#### Software

All analyses will be conducted using SAS version 9.4. R will likely be used to combine the results from each province (see the combining outcome results from Alberta and Ontario section below).

#### Descriptive Statistics

Categorical measures will be summarized as frequencies and percentages, and continuous variables as means and standard deviations (SD) or medians and interquartile ranges (IQR) as appropriate. Baseline characteristics will be categorized by baseline eGFR category (≥60, 45–59, and <45 mL/min per 1.73 m^2^). Differences in baseline characteristics between the pregnant and non-pregnant groups will be examined using standardized differences, with differences ≥10% considered meaningful.^25^

As described, we will use inverse probability of treatment weighting on the propensity score to balance baseline characteristics between the pregnant and non-pregnant groups. Methods to account for the weighting will be used in all comparative analyses, and bootstrapping will be used to create confidence intervals (except for outcomes where too few events in one or both groups prevent the analysis).

### Objective 1: Rate of Long-term Progressive Kidney Disease

The relationship between pregnancy and the rate of progressive kidney disease will be analyzed using a weighted cause-specific hazard model, accounting for the competing risk of death and expressed as a weighted HR with 95% confidence intervals. The primary outcome is the time to progressive kidney disease, incorporating pregnancy group and eGFR category indicators and an interaction term (eGFR category * pregnancy status) to assess effect modification. The proportional hazards assumption will be tested using Schoenfeld residuals and log-log survival curves. The primary focus is on assessing additive interaction. The parameters from these models will be used to assess additive interaction; we will obtain estimates across eGFR strata for the RERI, the proportion attributable to interaction, the synergy index, and a measure of interaction on the multiplicative scale.^26-30^ Additive interaction will be assessed using RERI obtained from hazard ratios.^31^ Testing RERI = 0 will be the primary statistical test, where an RERI = 0 indicates no additive interaction.

We will present the cause-specific HRs stratified by the 3 eGFR categories. We will also visualize the long-term risk by graphing weighted CIF curves for up to 10 years of follow-up for pregnant and non-pregnant females stratified by the 3 eGFR categories.

If interaction exists for the primary outcome across the 3 eGFR categories, we will illustrate the results with baseline eGFR as a continuous variable using restricted cubic splines.

### Objective 2: Annualized Change in Estimated Glomerular Filtration Rate

This will be analyzed using linear mixed-effects models with random, individual-specific intercepts with an unstructured covariance to account for repeated measures within the same individual. The model will include fixed effects or indicators for the pregnancy group, eGFR category, and follow-up time, as well as their 3-way interaction (eGFR category * indicator for pregnancy [pregnant/non-pregnant] * follow-up time).

### Objective 3: Time-to-Event Analysis Stratified by Proteinuria Category

If there are enough proteinuria tests, we will repeat the analysis described for objective 1 but now add the proteinuria category (normal/mild, moderate, or severe) as a 3-way interaction with the pregnancy group and eGFR category. Those with missing baseline proteinuria will be excluded from this analysis.

### Combining Outcome Results From Ontario and Alberta

We will conduct a 2-province distributed analysis for the first and third objectives using inverse probability weighted (IPW) Cox regression analyses across both provinces. This will be a privacy-preserving method based on summary-level data which produces the same estimated hazard ratio as from a combined individual-level data analysis.^32^ This method relies on 4-column risk-set tables, which summarize: total weights of treated events, total weights of all events, total weights of treated patients, and total weights of untreated patients.

Each province will independently calculate these summary tables using their individual-level data. These summary tables will need to be produced within each eGFR strata, to estimate stratum-specific hazard ratios which can then be used to estimate the RERI. To facilitate variance estimation of the hazard ratios and the RERI, the summary tables above will be reproduced in 200 resampled bootstrap samples. Within each province, the propensity scores and weights will be re-estimated within each bootstrap sample before the summary table above is also reproduced.

The generated risk-set tables will be shared in a single data transfer step to minimize complexity. The ICES will combine the summary-level data from both provinces to estimate the hazard ratios, RERI, and their 95% confidence intervals, enabling a robust and consistent analysis while maintaining data privacy and adhering to regulatory compliance.

#### Two-province meta-analysis

For the second objective, we will use a fixed-effects model to generate a pooled meta-analytic estimate across the 2 provinces. Our interaction test will determine whether there is a difference in the pooled estimates across the 3 eGFR categories. A fixed-effect rather than a random-effect model is often considered most appropriate when the estimate from each province is derived using the same design and methodology. A reasonable assumption in this study is that the effect size is common across provinces as they are in the same country, both have universal health care systems, and comparable population demographics are expected.^33^ Heterogeneity across provinces will be assessed using the I^2^ statistic, with a value exceeding 50% indicating high heterogeneity.

### Reporting Statistical Significance

When reporting the *P* values for the study, we will conduct a series of sequential tests as described below to control for multiple testing and family-wise type I error rate. We will continue reporting *P* values until no statistical significance is found (*P* ≥ .05). After that point, we will report 95% confidence intervals for effect estimates without adjusting for multiple statistical tests.

- First, we will test for additive interaction for objective 1.
- Next, we will test for additive interaction for objective 2.
- Finally, we will test for additive interaction for objective 3.

### Additional Analyses

We will conduct 5 additional analyses.

First, we will examine the separate components of the primary outcome using the same analytic technique used for the primary composite outcome.

Second, we will examine the weighted subdistribution HR of the primary composite outcome with death treated as a competing event.

Third, we will conduct an E-value analysis of the primary outcome to assess the effect of unmeasured confounders among those with an eGFR <45 mL/min per 1.73 m^2^. The analysis aims to determine the minimum strength of association on the HR scale that unmeasured confounders would need to have to negate an observed exposure-outcome association. A publicly available online E-value calculator will be used.^32-34^

Fourth, serum creatinine levels tend to decrease due to hyperfiltration caused by the pregnancy. As a result, eGFR levels during pregnancy can be unreliable. We will analyze the annualized change in eGFR, this time excluding all serum creatinine values in the year following the date of cohort entry (conception date or fake conception date). In addition, we will repeat the analysis, calculating the annualized change in eGFR starting a year after cohort entry, only including pregnant and non-pregnant individuals who survive to this point.

Fifth, to help with result interpretation, we will describe the percentage of females in each group and each eGFR category in follow-up who have medications dispensed by outpatient pharmacies that block the renin-angiotensin-aldosterone system.

### Study Size, Statistical Power and Minimizing Bias

Based on a feasibility analysis using ICES data from Ontario, we anticipate including over 400 000 pregnant females and 1.2 million non-pregnant females (eFigure 1 in Supplemental Appendix). This includes at least 395 000 pregnant females with eGFR ≥60 mL/min/1.73 m^2^, 300 with eGFR 45 to 59 mL/min/1.73 m^2^, and 110 with eGFR <45 mL/min/1.73 m^2^. Data from Alberta will add approximately 100 000 pregnant females and additional non-pregnant individuals. The Health Disease Research Network Data Access Support Hub (DASH) has confirmed feasibility in Alberta based on study criteria.

We anticipate the pregnant group in our study will have baseline characteristics similar to those in our feasibility analysis. Diabetes prevalence is expected to be 3% in individuals with eGFR >60 mL/min/1.73 m^2^ and 8% to 12% in those with eGFR <60 mL/min/1.73 m^2^. Hypertension prevalence is estimated at <5% for eGFR >60 mL/min/1.73 m^2^ and 20% to 30% for eGFR <60 mL/min/1.73 m^2^. Baseline nephrology visits in the 2 years before conception are expected to be low, with only 25% of those with eGFR 45 to 59 mL/min/1.73 m^2^ and 63% of those with eGFR <45 mL/min/1.73 m^2^ having at least 1 nephrologist visit.

The power calculation for this protocol was informed by the feasibility study conducted in the Ontario population at ICES (see eFigure 1 in Supplemental Appendix). With a 2-sided 5% significance level, we will have 80% power to detect additive interaction for the primary outcome using the RERI on the risk ratio (RR) scale.^35,36^ The power calculation available for testing additive interactions is limited to binary outcomes when comparing the interaction between variables with 2 categories each in an unmatched setting. There are some differences in the planned analysis, as we will have 3 eGFR groups rather than 2. For this reason, we used a conservative eGFR cut point of 60 mL/min per 1.73 m^2^ to avoid over-estimating the effect size we would observe when comparing females with an eGFR <45 mL/min per 1.73 m^2^ to females with an eGFR ≥60 mL/min per 1.73 m^2^ (ie, the groups of females with the highest and lowest rate of progressive CKD, respectively). Furthermore, rather than a binary outcome, we have a time-to-event outcome, which consists of a continuous event time and a binary censoring indicator. Previous work has shown that you can fit a logistic model to survival data, but there is some information loss, particularly when there is variable follow-up time. As such, we expect to gain some statistical efficiency by using a weighted cause-specific hazard model rather than a binary model.

In a 2-group retrospective cohort study, differences in baseline health between groups can complicate outcome interpretation due to residual confounding from measured and unmeasured factors. For instance, healthier females may be more likely to conceive, making the non-pregnant group inherently less healthy. To address this, we will use restriction and propensity score weighting to balance the groups on measured baseline characteristics and minimize confounding.^11-14^ Finally, we will use E-value analysis to help quantify the influence any unmeasured confounding may have on effect estimates.

As serum creatinine measurements will follow routine care rather than a research protocol, we will compare the rate of testing by group within each eGFR category to assess potential surveillance bias. The risk of bias will be evaluated using the ROBINS-I tool, covering 7 domains: confounding, sample selection, exposure classification, deviations from exposure, missing data, outcome measurement, and result reporting.^37^ Two independent reviewers will assess and report the risk of bias, which is anticipated to be low.

## Discussion

This protocol describes the design and statistical analysis plan for a population-based cohort study to assess the impact of baseline kidney function on the rate of progressive kidney disease after pregnancy. We expect our study will contain at least 400 000 females in the pregnant group and 1.2 million females in the non-pregnant group before statistical weighting, including more than 400 individuals with a pre-conception eGFR <60 mL/min per 1.73 m^2^. We expect the median follow-up in each group to be 5 years (maximum 17 years). To our knowledge, this will be one of the largest sample sizes to assess the study question to date.^4^

We designed this study to produce accurate estimates with minimal bias. The study’s strengths include a population-based design, the use of pre-conception eGFR, and the inclusion of individuals from 2 Canadian provinces. Our goal is to achieve results that are reliable and generalizable. We anticipate a low rate of lost follow-up, which would only occur if an individual becomes ineligible for provincial health care or moves out of the province (<0.5% per year in Ontario and <1.5% per year in Alberta).^38^ We will evaluate and report the potential for bias in the study using the ROBINS-I tool. Our goal is to provide more precise estimates compared to previous studies, which will better assist health care providers in advising those with kidney disease who are considering pregnancy. Our findings will also inform guidelines and policies regarding the counseling, care, and long-term monitoring of individuals with kidney disease who become pregnant.

Our study relies on administrative data and has some associated limitations. Kidney function will be measured as part of routine care at baseline and in follow-up rather than according to a research schedule. We anticipate some missing data for baseline proteinuria and baseline medications. However, the remaining study variables should have minimal missing values. We acknowledge health care codes do not perfectly capture all variables and outcomes (usually, the codes are highly specific and moderately sensitive).^39^ Yet, they provide generally applicable measures for Canadian residents, and we have purposefully selected study variables that can be defined using validated codes and algorithms. Some potential confounders (eg, body weight and blood pressure) will not be available in our data sources. Consequently, we will describe our findings in terms of associations rather than causal relationships.

There is the potential for immortal time bias and ascertainment bias, as the pregnant individuals must have survived to at least 20 weeks’ gestation after conception to be eligible for inclusion in the study. However, data suggest maternal mortality prior to 20 weeks’ gestation is exceedingly low.^40^

In our previous study, we discovered that pre-eclampsia affected 25% of pregnancies in the eGFR category <45 mL/min/1.73 m^2^, but only 6% of pregnancies with eGFR >60 mL/min/1.73 m^2^.^24^ Pre-eclampsia is a known risk factor for progressive kidney decline. However, in this study, it would be challenging to conduct mediation analyses to isolate its specific impact from other contributors to kidney function decline. In addition, we are not considering whether those in the pregnancy group have a higher likelihood of primary kidney disease recurrence than the non-pregnant group.

Out-of-hospital deliveries are not included in this study, although we do not anticipate this will meaningfully impact the generalizability of results (less than 1% of deliveries occur at home in Ontario and less than 3.5% in Alberta). We do not have specific administrative codes for the use of reproductive technologies in this study; however, females who are biologically unable to conceive will not be accrued. Owing to privacy regulations, we will not be able to report rare events that occur in fewer than 6 individuals. We also do not have accurate indicators for race or ethnicity in our provincial administrative databases.

Finally, we will not be able to assess the impact of serial pregnancies on the risk of progressive kidney disease due to our study design and accrual choices.

## Conclusion

Better information is needed about how baseline kidney function may impact the progression of kidney disease after pregnancy. This study will determine whether the baseline eGFR level and proteinuria influence the rate of progressive kidney disease after pregnancy and will provide data on the cumulative incidence in pregnant and non-pregnant individuals across 3 categories of baseline eGFR. It will be the largest study on this topic, and the results will provide valuable insights regarding the long-term risks of pregnancy. The findings will also support future efforts to create accurate risk-estimation tools to predict post-pregnancy renal prognosis to provide personalized counseling.

## Supplemental Material

sj-docx-1-cjk-10.1177_20543581251318836 – Supplemental material for Impact of Baseline Kidney Function on the Rate of Progressive Kidney Disease After Pregnancy: A Population-Based Cohort Study Research Protocol Supplemental material, sj-docx-1-cjk-10.1177_20543581251318836 for Impact of Baseline Kidney Function on the Rate of Progressive Kidney Disease After Pregnancy: A Population-Based Cohort Study Research Protocol by Lavanya Bathini, Nivethika Jeyakumar, Jessica Sontrop, Eric McArthur, Yuguang Kang, Bin Luo, Aminu Bello, David Collister, Sofia Ahmed, Padma Kaul, Erik Youngson, Branko Braam, Nir Melamed, Michelle Hladunewich and Amit X. Garg in Canadian Journal of Kidney Health and Disease
