# Supplementary material for: Impact of Baseline Kidney Function on the Rate of Progressive Kidney Disease After Pregnancy: A Population-Based Cohort Study Research Protocol
Source: Can J Kidney Health Dis. 2025 Feb 28;12:20543581251318836. doi: 10.1177/20543581251318836 (PMC11869263; doi:10.1177/20543581251318836)
Supplement: sj-docx-1-cjk-10.1177_20543581251318836 – Supplemental material for Impact of Baseline Kidney Function on the Rate of Progressive Kidney Disease After Pregnancy: A Population-Based Cohort Study Research Protocol [file sj-docx-1-cjk-10.1177_20543581251318836.docx]

**Appendix**

**Table of Contents**

eTable 1. Checklist of recommendations for reporting of observational studies using the REporting of studies Conducted using Observational Routinely-collected health Data (RECORD) Statement………………………………………..2

eFigure 1. Sample flow from feasibility analysis..……………………………………………….….…..6

eTable 2. Administrative data codes used to define baseline characteristics…………….…7

eTable 3. Assumptions for power calculations…………………………………………………………….18

**eTable 1**. **Checklist of recommendations for reporting of observational studies using the REporting of studies Conducted using Observational Routinely-collected health Data (RECORD) Statement**

|  | **Item No** | | | **STROBE items** | | **RECORD items** | | **Reported** | | |
| --- | --- | --- | --- | --- | --- | --- | --- | --- | --- | --- |
| **Title and abstract** | 1 | | | (a) Indicate the study's design with a commonly used term in the title or the abstract.  (b) Provide in the abstract an informative and balanced summary of what was done and what was found. | | (1.1) The type of data used should be specified in the title or abstract. When possible, the name of the databases used should be included.  (1.2) If applicable, the geographic region and time frame within which the study took place should be reported in the title or abstract.  (1.3) If linkage between databases was conducted for the study, this should be clearly stated in the title or abstract. | | Abstract | | |
| **INTRODUCTION** | | | | | | | | | | |
| Background/ rationale | | 2 | Explain the scientific background and rationale for the investigation being reported. | | | |  | | | Introduction |
| Objectives | | 3 | State specific objectives, including any prespecified hypotheses. | | | |  | | | Introduction |
| **METHODS** | |  |  | | | |  | | |  |
| Study design | | 4 | Present key elements of study design early in the paper. | | | |  | | | Methods: Study Design |
| Setting | | 5 | Describe the setting, locations, and relevant dates, including periods of recruitment, exposure, follow-up, and data collection. | | | |  | | | Methods: Study Design |
| Participants | | 6 | (a) Give the eligibility criteria, and the sources and methods of selection of participants. Describe methods of follow-up.  (b) For matched studies, give matching criteria and number of exposed and unexposed. | | | | (6.1) The methods of study population selection (such as codes or algorithms used to identify subjects) should be listed in detail. If this is not possible, an explanation should be provided.  (6.2) Any validation studies of the codes or algorithms used to select the population should be referenced. If validation was conducted for this study and not published elsewhere, detailed methods and results should be provided.  (6.3) If the study involved linkage of databases, consider use of a flow diagram or other graphical display to demonstrate the data linkage process, including the number of individuals with linked data at each stage. | | | Methods: Participants, Matching |
| Variables | | 7 | Clearly define all outcomes, exposures, predictors, potential confounders, and effect modifiers. Give diagnostic criteria, if applicable. | | | | (7.1) A complete list of codes and algorithms used to classify exposures, outcomes, confounders, and effect modifiers should be provided. If these cannot be reported, an explanation should be provided. | | | Methods: Study Exposure, Renal Outcomes |
| Data sources/   measurement | | 8 | For each variable of interest, give sources of data and details of methods of assessment (measurement). Describe comparability of assessment methods if there is more than one group. | | | |  | | | Methods: Data Sources  Supplemental Table 2 |
| Bias | | 9 | Describe any efforts to address potential sources of bias. | | | |  | | | Statistical analysis Plan: Study Size and Bias |
| Study size | | 10 | Explain how the study size was arrived at. | | | |  | | | Methods: Population  eFigure 1 |
| Quantitative variables | | 11 | Explain how quantitative variables were handled in the analyses. If applicable, describe which groupings were chosen and why. | | | |  | | | Methods: Statistical Analyses |
| Statistical methods | | 12 | (a) Describe all statistical methods, including those used to control for confounding.  (b) Describe any methods used to examine subgroups and interactions.  (c) Explain how missing data were addressed.  (d) If applicable, explain how loss to follow-up was addressed.  (e) Describe any sensitivity analyses. | | | |  | | | Methods: Statistical Analyses |
| Data access and cleaning methods | |  | N/A | | | | (12.1) Authors should describe the extent to which the investigators had access to the database population used to create the study population.  (12.2) Authors should provide information on the data cleaning methods used in the study. | | | Methods: Data Sources  Data Access/Access to Data Analysis Protocol |
| Linkage | |  | N/A | | | | (12.3) State whether the study included person-level, institutional-level, or other data linkage across two or more databases. The methods of linkage and methods of linkage quality evaluation should be provided. | | | Methods: Data Sources |
| **RESULTS** | |  |  | | | |  | | |  |
| Participants | | 13 | (a) Report numbers of individuals at each stage of study--e.g. numbers potentially eligible, examined for eligibility, confirmed eligible, included in the study, completing follow-up, and analyzed.  (b) Give reasons for non-participation at each stage.  (c) Consider use of a flow diagram. | | | | (13.1) Describe in detail the selection of the persons included in the study (i.e., study population selection), including filtering based on data quality, data availability, and linkage. The selection of included persons can be described in the text and/or by means of the study flow diagram. | | | Results: Baseline Characteristics (Table 1)  Figure 1 |
| Descriptive data | | 14 | (a) Give characteristics of study participants (e.g. demographic, clinical, social) and information on exposures and potential confounders.  (b) Indicate number of participants with missing data for each variable of interest.  (c) Summarize follow-up time (e.g. average and total amount). | | | |  | | | Results: Baseline Characteristics  Table 1 |
| Outcome data | | 15 | Report numbers of outcome events or summary measures over time. | | | |  | | | Results: Renal Outcomes |
| Main results | | 16 | (a) Give unadjusted estimates and, if applicable, confounder-adjusted estimates and their precision (e.g. 95% confidence interval). Make clear which confounders were adjusted for and why they were included.  (b) Report category boundaries when continuous variables were categorized.  (c) If relevant, consider translating estimates of relative risk into absolute risk for a meaningful time period. | | | |  | | | Results |
| Other analyses | | 17 | Report other analyses done (e.g. analyses of subgroups and interactions, and sensitivity analyses). | | | |  | | | Results: Renal Outcomes Proteinuria Outcomes by eGFR and Proteinuria Severity |
| Key results | | 18 | Summarize key results with reference to study objectives. | | | |  | | | Discussion |
| Limitations | | 19 | Discuss limitations of the study, taking into account sources of potential bias or imprecision. Discuss both direction and magnitude of any potential bias. | | | | (19.1) Discuss the implications of using data that were not created or collected to answer the specific research question(s). Include discussion of misclassification bias, unmeasured confounding, missing data, and changing eligibility over time, as they pertain to the study being reported. | | | Discussion |
| Interpretation | | 20 | Give a cautious overall interpretation of results considering objectives, limitations, multiplicity of analyses, results from similar studies, and other relevant evidence. | | | |  | | | Discussion |
| Generalizability | | 21 | Discuss the generalizability (external validity) of the study results. | | | |  | | | Discussion |
| **OTHER INFORMATION** | | | | | | | | | | |
| Funding | 22 | | | Give the source of funding and the role of the funders for the present study and, if applicable, for the original study on which the present article is based. | |  | | Funding | | |
| Accessibility of protocol, raw data, and programming code | |  | N/A | | (22.1) Authors should provide information on how to access any supplemental information such as the study protocol, raw data, or programming code. | | | | Data access/access to data analysis protocol | |

**eFigure 1.** Sample flow from feasibility analysis

Abbreviations: eGFR, estimated glomerular filtration rate; RPDB, Registered Persons Database.

1 If the most recent index serum creatinine test was an inpatient test [ER or hospitalization] and if the corresponding eGFR differed from the most recent outpatient test by 10 mL/min/1.73 m^2^or more, this record was excluded.   Note: eGFR will be assessed using the race free equation, during the period of study all serum creatinine testing in the province was traceable to isotope dilution mass spectrometry (IDMS), and we have previously demonstrated in our province that outpatient serum creatinine measurements done on a single occasion represent stable values.

2 Estimate date of conception was computed using gestational weeks at delivery in the MOMBABY database and is defined as:[B_BDATE] minus [B_GESTWKS_DEL]*7

3 For females with multiple pregnancies during our study period, we restricted the final cohort to only include the last pregnancy.

4 Pregnancies were randomly selected and we randomly assigned following the same distribution of days between serum creatinine tests and conception dates to a control serum creatinine test in the control cohort to derive a pseudo-conception date. We then used the same distribution of days between conception date and delivery date in the selected pregnant to assign a pseudo-delivery date in the control record.

5 For females with multiple control records during our study period, we randomly selected one record per female in the final cohort.

**eTable 2.** Administrative data codes used to define baseline characteristics and other study variables

| **Alberta Database** | **Ontario Database** | **Variable** | **Code or Algorithm** |
| --- | --- | --- | --- |
| ***Age*** | | | |
| AHCIP | RPDB | AGE | N/A |
| ***Neighbourhood income quintile*** | | | |
| LDP | RPDB | INCQUINT | N/A |
| ***Rural residence*** | | | |
| LDP | RPDB | RURAL | N/A |
| ***Dependency*** | | | |
| LDP | ONMARG | DEPENDENCY_Q_DA | N/A |
| ***Material deprivation*** | | | |
| LDP | ONMARG | DEPRIVATION_Q_DA | N/A |
| ***Residential instability*** | | | |
| LDP | ONMARG | INSTABILITY_Q_DA | N/A |
| ***Location of index serum creatinine test*** | | | |
| PROV LAB  CIHI-DAD/NACRS | OLIS  CIHI-DAD/NACRS | OBSERVATIONCODE  ADMDATE  DDATE  REGDATE  DISPDATE | 14682-9 |
| ***Prior Pregnancies*** | | | |
| APHP | MOMBABY | M_PREVBIRTH_DERIVED  M_PREVBIRTH_ORIGINAL  M_IKN  BDATE |  |
| ***Days between most recent prior pregnancy and index pregnancy*** | | | |
| APHP | MOMBABY | M_PREVBIRTH_DERIVED  M_PREVBIRTH_ORIGINAL  M_IKN  BDATE |  |
| ***Multiple gestation at index*** | | | |
| APHP | MOMBABY | M_MULTIBIRTH |  |
| ***Delivery type at index*** | | | |
| CIHI-DAD | CIHI-DAD | CCI | Vaginal Delivery  5MD50, 5MD51, 5MD52  Caesarean Delivery  5MD60  Forceps/Vacuum Delivery  5MD53,5MD54, 5MD55  Breach Delivery  5MD56 |
| Practitioner Claims | OHIP | Fee code | Vaginal Delivery  P006, P020  Caesarean Delivery  P018, P041, P042 |
| ***Gestational diabetes*** | | | |
| CIHI-DAD | CIHI-DAD | ICD10 | O24 |
| ***Gestational hypertension*** | | | |
| CIHI-DAD | CIHI-DAD | ICD10 | O13009, O13004, O13003, O13002, O13001 |
| ***HELLP Syndrome*** | | | |
| CIHI-DAD | CIHI-DAD | ICD10 | O14209, O14204, O14203, O14202, O14201 |
| ***Placenta Previa*** | |  |  |
| CIHI-DAD | CIHI-DAD | ICD10 | O44 |
| ***Placenta Abruption*** | | | |
| CIHI-DAD | CIHI-DAD | ICD10 | O45 |
| Practitioner Claims | OHIP | Diagnostic code | 641, 043 |
| ***Pre-eclampsia, eclampsia*** | | | |
| CIHI-DAD | CIHI-DAD | ICD10 | O14909, O14904, O14903, O14902, O14901, O14209, O14204, O14203, O14202,  O14201, O14109, O14104, O14103, O14102, O14101, O14009, O14004, O14003,  O14002, O14001, O15909, O15204, O15202, O15103, O15102, O15101, O15003, O15002, O15001, O11 |
| Practitioner Claims | OHIP | Diagnostic code | 642 |
| ***Pre-mature rupture of membrane*** | | | |
| CIHI-DAD | CIHI-DAD | ICD10 | O42 |
| ***Postpartum hemorrhage*** | | | |
| CIHI-DAD | CIHI-DAD | ICD10 | O72 |
| Practitioner Claims | OHIP | Diagnostic code | 666 |
| ***Coagulopathy*** | | | |
| CIHI-DAD | CIHI-DAD | ICD10 | D686, I26, I260, I269, I801, I802, I803, I822, I828, I829, O87102, O87104, O87109, O87802, O87804, O87809, O87902, O87904, O87909, O88201, O88202, O88203, O88204, O88209 |
|  |  | CCI | 3GT20WC, 3GT20WE, 3GT70CA, 3GT70CC, 3GT70CE, 3GT70KC, 3GT70KD, 3GT70KE, 3IM10VC, 3IM10VX, 3IM10VY, 3IM12VA, 3JY10VA, 3JY10VC, 3JY10VN, 3JY10VX, 3JY12VA, 3JY20WC, 3JY20WE, 3KR10VA, 3KR10VC, 3KR10VN, 3KR12VA, 3KX10VA, 3KX10VC, 3KX10VN, 3KX10VX, 3KX12VA, 3KX30DA, 3KX30DB, 3KX30DC, 3KX30DD |
| Practitioner claims | OHIP | Diagnostic codes | 415, 451, 671, 677 |
|  |  | Fee codes | J193, J198, J202, J493, J498, J502, J659, J660, J859, J860, X125, X406, X407 |
| ***Acute kidney injury*** | | | |
| CIHI-DAD | CIHI-DAD | ICD10 | N17 |
| ***Autoimmune disease*** | | | |
| CIHI-DAD | CIHI-DAD | ICD10 | E05, E063, G35, L40, M070, M071, M072, M073, M090, M05, M06, M350, M320,  M321, M328, M329, M300, M308, M311, M313, M3130, M3131, M317, M318, M319, D891, K50, K51 |
| Practitioner Claims | OHIP | Diagnostic code | 714, 555, 556 |
| ***History of Cancer*** | | | |
| CIHI-DAD | CIHI-DAD | ICD10 | 971, 980, 982, 984, 985, 986, 987, 988, 989, 990, 991, 993, C15, C18, C19, C20, C22, C25, C34, C50, C56, C61, C82, C83, C85, C91, C92, C93, C94, C95, D00, D05, D010, D011, D012, D022, D075 |
| Practitioner Claims | OHIP | Diagnostic code | 203, 204, 205, 206, 207, 208, 150, 154, 155, 157, 162, 174, 175, 183, 185 |
| ***Cardiovascular disease*** | | | |
| CIHI-DAD | CIHI-DAD | ICD10 | I20, I21, I22, I23, I24, I25,E780, Z955, Z958, Z959, R931, T822, I700, I702, I708, I709, I731, I738, I739, K551, I60, I600, I601, I602, I603, I604, I605, I606, I607, I608, I609, I61, I610, I611, I612, I613, I614, I615, I616, I618, I619, I630, I631, I632, I633, I634, I635, I638, I639, I64, H341, G450, G451, G452, G453, G458, G459, H340, I099, I255, I420, I426,I425, I427, I428, I429, I43, I430, I431, I432, I438, I50, I500, I501 |
|  |  | CCI | 1IJ26, 1IJ27, 1IJ50, 1IJ54, 1IJ57, 1IJ76, 1KA76, 1KA50, 1KE76, 1KG50, 1KG57, 1KG76MI, 1KG87, 1IA87LA, 1IB87LA, 1IC87LA, 1ID87, 1KA87LA, 1KE57, |
| Practitioner Claims | OHIP | Diagnostic code | 410, 412, 413, 436, 432, 435,428 |
|  |  | Fee code | R741, R742, R743, G298, E646, E651, E652, E654, E655, G262, Z434, Z448, R787, R780, R797, R804, R809, R875, R815, R936, R783, R784, R785, E626, R814, R786, R937, R860, R861, R855, R856, R933, R934, R791, E672, R794, R813, R867, E649, R701, R702, Z429 |
| ***Chronic Dialysis*** | | | |
| CIHI-DAD | CIHI-DAD | CCI | 1PZ21 |
| Practitioner Claims | OHIP | Fee code | R849, G323, G325, G326, G860, G862, G865, G863, G866, G330, G331, G333, G861, G082, G083, G085, G090, G091, G092, G093, G094, G095, G096, G294, G295, G864, H540, H740 |
| ***Cystic kidney disease*** | | | |
| CIHI-DAD | CIHI-DAD | ICD10 | Q611, Q612, Q613 |
| Practitioner Claims | OHIP | Diagnostic code | 753,593 |
| ***Death*** | | | |
| Vital Statistics | RPDB | DTHDATE |  |
| ***Diabetes*** | | | |
| LDP | ODD | DIAGDATE |  |
| ***Dialysis*** | | | |
| CIHI-DAD | CIHI-DAD | ICD10 | T824, Y602, Y612, Y622, Y841, Z49, Z992 |
|  |  | CCI | 1PZ21,1OT53DATS,1OT53HATS,1OT53LATS,1SY55LAFT,7SC59QD,1KY76,1KG76MZXXA,1KG76MZXXN,1JM76NC,1JM76NCXXN, |
| Practitioner Claims | OHIP | Fee code | R850, G324, G336, G327, G862, G865, G099, R825, R826, R827, R833, R840, R841, R843, R848, R851, R946, R943, R944, R945, R941, R942, Z450, Z451, Z452, G864,  R852, R853, R854, R885, G333, H540, H740, R849, G323, G325, G326, G860, G863, G866, G330, G331, G861, G082, G083, G085, G090, G091, G092, G093, G094, G095,  G096, G294, G295 |
| ***Hypertension*** | | | |
| LDP | HYPER | DIAGDATE |  |
| ***GN Testing*** | | | |
| PROVLAB | OLIS | OBSERVATIONCODE | 17351-8, 5128-4, 10676-5, 10900-9, 11258-1, 11259-9, 13126-8, 13248-0, 13294-4, 13950-1, 13951-9, 13952-7, 13953-5, 13954-3, 13955-0, 16128-1, 16129-9, 16933-4, 16935-9, 20416-4, 20442-0, 20571-6, 20575-7, 22312-3, 22314-9, 22315-6, 22316-4, 22319-8, 22320-6, 22321-4, 22322-2, 22327-1, 22330-5, 24113-329609-529610-3  31204-1, 31844-4, 32018-4, 32286-7, 32366-7, 35680-8, 40724-7, 40727-0, 41996-0, 42595-9, 51459-6, 5179-7, 5181-3, 5185-4, 5189-6, 5191-2, 51913-2, 51914-0, 5193-8, 5195-3, 5196-1, 5198-7, 5200-1, 58936-6, 65633-0, 7905-3, 10676-5, 11259-9, 11502-2, 13955-0, 16128-1, 16129-9, 19146-0,20416-4,20571-6,22327-1,29609-5,30896-5,32286-7,41852-5, 41996-0,48574-8,48575-5, 49372-6, 5010-4, 5012-0, 5198-7, 56929-3, 57006-9, XON10296-2, XON10312-7, XON10313-5, XON10442-2, XON12338-0, XON12663-1, XON13033-6, XON13050-0 |
| CIHI-DAD | CIHI-DAD | ICD10 | B16, B170, B180, B181, B171, B182, B20, B21, B22, B23, B24 |
| Practitioner Claims | OHIP | Fee code | L944, G032, L319, L919 |
|  |  | Diagnostic code | 042, 043, 044 |
| Female Hormonal Testing | | | |
| Practitioner Claims | OHIP | OBSERVATIONCODE | L087, L088, L089, L090, L091, L207, L205, L206, L315, L310, L311, L312, L313, L331, L333, G015, G019, G018, G020, G026, L328, L332, G017 |
| TSH Testing | | | |
| Provlab | OLIS | OBSERVATIONCODE | 11579-0, 11580-8, 14297-6, 14920-3, 14928-6, 3015-5, 3016-3 |
| Practitioner claims | OHIP | Fee code | G016 |
| B-HCG Testing (urine and serum) | | | |
| Provlab | OLIS | OBSERVATIONCODE | 19080-1, 20994-0, 2106-3, 2107-1, 2110-5, 2111-3, 2112-1, 2118-8, 2119-6, 21198-7, 25372-4, 45194-8 |
| Practitioner Claims | OHIP | Fee code | L318, G021 |
| Blood Type and Screen | | | |
| Provlab | OLIS | OBSERVATIONCODE | 10331-7, 882-1, 883-9, 14577-1, 14578-9, 14580-5, 14906-2, 14908-8, 19057-9, 34474-7, 34530-6, 44086-7, 46268-9, 46270-5, 51892-8, 54417-1, 57743-7, 884-7, XON12168-1, XON12365-3 |
| Group B Strep Tests | | | |
| Practitioner Claims | OHIP | Fee Code | L636, L638, L640, L659, Q673, G014 |
| STI Testing | | | |
| Practitioner Claims | OHIP | Fee code | L622, L669, L625 |
| Pap Smear | | | |
| Practitioner Claims | OHIP | Fee Code | G394, E430, Q001, Q011, Q105, Q106, Q107, Q108, Q109, Q681, Q140 |
| Urine Culture | OHIP | Fee Code | L253, L254, L255, L633, L634, L641, G009, G010 |
| Gynecologic Diagnostic Imaging |  |  |  |
| ***Genitourinary disease*** | | | |
| CIHI-DAD | CIHI-DAD | ICD10 | N00, N01, N02, N03, N04, N05, N06, N07, N08, N09, N10, N11, N12, N13, N14, N15, N16, N17, N18, N19, N20, N21, N22, N23, N24, N25, N26, N27, N28, N29, N30, N31, N32, N33, N34, N35, N36, N37, N38, N39, N40, N41, N42, N43, N44, N45, N46, N47, N48, N49, N50, N51, N52, N53, N54, N55, N56, N57, N58, N59, N60, N61, N62, N63, N64, N65, N66, N67, N68, N69, N70, N71, N72, N73, N74, N75, N76, N77, N78, N79, N80, N81, N82, N83, N84, N85, N86, N87, N88, N89, N90, N91, N92, N93, N94, N95, N96, N97, N98, N99 |
| ***Urinary tract infection*** | | | |
| CIHI-DAD | CIHI-DAD | ICD10 | N10, N11, N110, N111, N118, N119, N12, N136, N151, N159, N160, N300, N308, N309, N340, N390, N410, N411, N412, N413, N431, N45, N4500, N4501, N4502, N4590, N4591, N4592, O23901, O23902, O23903, O23904, O23909, O86202, O86204, O86209, O86302, O86304, O86309, T835, |
| ***Anxiety or Depression*** | | | |
| CIHI-DAD | CIHI-DAD | ICD10 | F063, F064, F204, F313, F314, F315, F32, F320, F321, F322, F323, F328, F329, F33, F330, F331, F332, F333, F334, F338, F339, F341, F400, F401, F402, F408, F409, F410, F411, F412, F413, F418, F419, F420, F421, F422, F428, F429, F430, F431, F432. |
| Practitioner Claims | OHIP | Diagnostic code | 311 |
| ***Disorders causing infertility*** | | | |
| CIHI-DAD | CIHI-DAD | ICD10 | N70, N700, N701, N709, N71, N710, N711, N719, N72, N73, N730, N731, N732, N733, N734, N735, N736, N738, N739, N74, N740, N741, N742, N743, N744, N748, N75, N750, N751, N758, N759, N76, N760, N761, N762, N763, N764, N765, N766, N768, N7680, N7688, N77, N770, N771, N778, N81, N810, N811, N812, N813, N814, N815, N816, N818, N819, N82, N820, N821, N822, N823, N824, N825, N828, N829, N83, N830, N831, N832, N833, N834, N8350, N8351, N8352, N836, N837, N838, N839, N84, N840, N841, N842, N843, N848, N849, N85, N850, N851, N852, N853, N854, N855, N856, N857, N858, N859, N86, N87, N870, N871, N872, N879, N88, N880, N881, N882, N883, N884, N888, N889, N89, N890, N891, N892, N893, N894, N895, N896, N897, N898, N899, N90, N900, N901, N902, N903, N904, N905, N906, N907, N908, N909, N91, N97, N970, N971, N972, N973, N974, N978, N979, N994, N92, N920, N921, N922, N923, N924, N925, N926, 88900, 88901, 88903, 88910, 88913, 88920, 88930, E65, N911, N80, N800, N801, N802, N803, N804, N805, N806, N808, N809, E06, E060, E061, E062, E063, E064, E065, E069, E07, E070, E071, E0781, E0788, E079, E230, E231, E232, E233, E236, E237, E012, E018, E02, E03, E030, E031, E032, E033, E034, E035, E038, E039, E05, E050, E051, E052, E053, E054, E055, E058, E059, E06, E060, E061, E062, E063, E064, E065, E069, E07, E070, E071, E0781, E0788, E079, E890, N950, N951, N952, E283, E282 |
|  |  | ICD9 | 6271, 6272, 6273, 6278, 6279,2563 |
|  |  | CCI | 1RB26BAEB, 1RB26BAEC, 1RB26DAEB, 1RB26DAEC, 1RB26HAEB, 1RB26LAEB, 1RB26LAEC, 1RB27JA, 1RB27JX, 1RB55DAEB, 1RB55DAEC, 1RB55LAEB, 1RB55LAEC, 1RF51FJFF, 1RF51FJGE, 1RF51FJLV, 1RF51LAAL, 1RF51LAFA, 1RF51LAFF, 1RF51LALV, 1RF52BA, 1RF52DA, 1RF55DAFF, 1RF55DAKR, 1RF55DANR, 1RF55DAPM, 1RM26BA, 1RM26BAEB, 1RM26BAEC, 1RM26CA, 1RM26DA, 1RM26DAEC, 1RM26HA, 1RM26HAEB, 1RM26LA, 1RM26LAEB, 1RM26LAEC, 1RM27JA, 1RM27JX, 1RM53BAEM, 1RM53CAEM, 1RM53DAEM, 1RM53HAEM, 1RM53LAEM, 1RM55BAEB, 1RM55CAEB |
| ***Gynecologic Surgeries*** | | | |
| CIHI-DAD | CIHI-DAD | CCP | 7700, 7710, 7711, 7712, 7719, 7720, 7730, 7740, 7741, 7742, 7750, 7751, 7752, 7760, 7761, 7762, 7763, 7769, 7770, 7780, 7781, 7782, 7789, 7790, 7791, 7792, 7793, 7794, 7795, 7799, 7800, 7810, 7820, 7821, 7822, 7830, 7831, 7832, 7839, 7840, 7841, 7842, 7849, 7850, 7851, 7852, 7853, 7859, 7860, 7861, 7862, 7863, 7864, 7869, 7870, 7880, 7881, 7889, 7890, 7891, 7892, 7893, 7894, 7895, 7896, 7897, 7899, 7900, 7910, 7920, 7921, 7922, 7923, 7929, 7930, 7940, 7950, 7951, 7952, 7959, 7980, 7981, 7982, 7989, 7990, 8100, 8101, 8109, 8120, 8121, 8129, 8130, 8131, 8132, 8133, 8139, 8140, 8150, 8151, 8152, 8159, 8160, 8161, 8169, 8170, 8180, 8190, 8191, 8192, 8193, 8194, 8195, 8196, 8197, 8198, 8199, 8200, 8210, 8211, 8212, 8213, 8214, 8220, 8221, 8222, 8223, 8230, 8240, 8241, 8242, 8243, 8250, 8251, 8252, 8260, 8261, 8262, 8263, 8264, 8269, 8270, 8280, 8281, 8282, 8283, 8289, 8290, 8291, 8292 |
|  |  | CCI | 1RB52BA, 1RB52CQ, 1RB52DA, 1RB52HA, 1RB52LA, 1RB56DA, 1RB56LA, 1RB57BA, 1RB57CQ, 1RB57DA, 1RB57HA, 1RB57LA, 1RB58DA, 1RB58LA, 1RB59DAAG, 1RB59DAAN, 1RB59DAGX, 1RB59LAAG, 1RB59LAAN, 1RB59LAGX, 1RB74DA, 1RB74LA, 1RB80DA, 1RB80LA, 1RB83DA, 1RB83LA, 1RB85DA, 1RB85LA, 1RB87DA, 1RB87LA, 1RB87RA, 1RB89DA, 1RB89LA, 1RB89RA, 1RD52BA, 1RD52DA, 1RD52LA, 1RD72DAAG, 1RD72DAGX, 1RD72LAAG, 1RD72LAGX, 1RD89DA, 1RD89LA, 1RD89RA, 1RF27JA, 1RF50BABJ, 1RF50BAGX, 1RF50BAKR, 1RF50BANR, 1RF50DA |
| ***Structural/Functional Kidney Disease (non-cystic)*** | | | |
| CIHI-DAD | CIHI-DAD | ICD10 | Q600, Q602, Q603, Q604, Q605, Q614, N13, N130, N131, N132, N133, N134, N135, N136, N137, N138, N139, N132, N20, N200, N201, N202, N209 |
|  |  | ICD9 | 5920, 5921, 5929 |
|  |  | CCI | 1PC58DAXXJ, 1PC58LBXXJ, 1PC58LBXXK, 1PC58PFXXJ, 1PC58PFXXK, 1PC58QPXXJ, 1PC58QPXXK, 1PC87DA, 1PC87LA, 1PC87LAXXE, 1PC87LAXXG, 1PC87NQ, 1PC89DA, 1PC89LB, 1PC89PF, 1PC89QF, 1PC91AB, 1PC91DA, 1PC91LB, 1PC91PF, 1PC91QF, 1PD89DA, 1PD89LB, 1PD89PF, 1PD89QF, 1PE57BAAM, 1PE57BAGX, 1PE57DTAG, 1PE57DTAM, 1PE57DTAS, 1PE57DTAZ, 1PE57DTBD, 1PE57DTGX, 1PE59BAAG, 1PE59BAAS, 1PE59BAAT, 1PE59BAAZ, 1PE59KQAP, 1PE59KQAQ, 1PE59KQAR, 1PG57BAAM, 1PG57BAGX, 1PG59BAAG, 1PG59BAAS, 1PG59BAAT, 1PG59BAAZ, 1PG59BAGX, 1PG59KQAP, 1PG59KQAQ, 1PG59KQAR |
| Practitioner Claims | OHIP | Fee code | S411, S412, S413, S415, S416, S420, S421, S423, S436, E760, E761, Z624, Z627, Z628, Z630 |
|  |  | Diagnostic code | 592 |
| ***Glomerular disease*** | | | |
| CIHI-DAD | CIHI-DAD | ICD10 | N00, N01, N02, N03, N04, N05, N06, N07, N08, E1020, E10200, E10201, E10202, E10203, E10204, E10209, E1021, E10210, E10211, E10212, E10213, E10214, E10219, E1120, E11200, E11201, E11202, E11203, E11204, E11209, E1121, E11210, E11211, E11212, E11213, E11214, E11219, E1320, E13200, E13201, E13202, E13203, E13204, E13209, E1420, E14200, E14201, E14202, E14203, E14204, E14209, E1421, E14210, E14211, E14212, E14213, E14214, E14219 |
| Practitioner Claims | OHIP | Diagnostic code | 580, 581 |
| ***Renal biopsy*** | | | |
| CIHI-DAD | CIHI-DAD | CCI | 1PC87, 2PC71BA, 2PC71DA, 2PC71HA, 2PC71LA, 2PE71BA, 2PE71DA, 2PE71HA, 2PE71LA |
|  |  | CCP | 6781, 6782 |
| Pracitioner Claims | OHIP | Fee Code | Z601, E820 |
| ***Renal transplant*** | | | |
| Practitioner Claims | OHIP | Fee code | S434, S435 |
| CORR | CORR | TREATMENT_CODE | 171 |
|  |  | TRANSPLANTED_ORGANN_TYPE_CODE | 10, 11, 12, 18, 19 |
| Practitioner Claims | OHIP | Fee code | Z601 |
| ***Sepsis*** | | | |
| CIHI-DAD | CIHI-DAD | ICD10 | A400, A401, A402, A403, A408, A409, A410, A411, A412, A413, A414, A4150, A4151, A4152, A4158, A4159, A4180, A4188, A419, R572, O85002, O85004, O85009 |
| Practitioner Claims | OHIP | Diagnostic code | 038 |
| ***Hospitalizations*** | | | |
| CIHI-DAD | CIHI-DAD | DDATE |  |
| ***Length of hospital stay*** | | | |
| CIHI-DAD | CIHI-DAD | ADMDATE | DDATE-ADMDATE |
|  |  | DDATE |  |
| ***Emergency department visits*** | | | |
| CIHI-DAD | CIHI-DAD | DDATE |  |
| ***Primary care physician visits*** | | | |
| Practitioner Claims | IPDB | MAINSPECIALTY | GP/FP |
|  | OHIP | SPEC | 00 |
|  |  | SERVDATE |  |
| ***Nephrology visits*** | | | |
| Practitioner Claims | IPDB | MAINSPECIALTY | NEPHROLOGY |
|  | OHIP | SPEC | 16 |
|  |  | Fee code | A160, A161, A163, A164, A165, A166, A168, A865, C160, C161, C162, C163, C164, C165, C166, C167, C169, C865, W165, W160, W865, W166, W862, W864, W867, W869, W164, W162, W161, W163, W168, A130, A131, A133, A134, A135, A136, A138, A435, C121, C122, C123, C124, C130, C131, C132, C133, C134, C135, C136, C137, C138, C139, C142, C143, C168, C435, C982, W121, W130, W131, W132, W133, W134, W138, W232, W234, W235, W236, W237W239, W435, W972, W982 |
|  |  | SERVDATE |  |
| ***Other Specialist Visits*** | | | |
| Practitioner Claims | IPDB | MAINSPECIALTY | OBSTETRICS AND GYNECOLOGY, INTERNAL MEDICINE, HEMATOLOGY, RHEUMATOLOGY, GASTROENTEROLOGY,CARDIOLOGY, ONCOLOGY,RESPIROLOGY,NEUROLOGY |
|  | OHIP | SPEC | 20 |
|  |  | SERVDATE |  |
| ***Gynecologic diagnostic imaging*** | | | |
| Diagnostic Imaging | OHIP | Fee code | J128, J135, J138, J149, J151, J157, J158, J159, J160, J161, J162, J428, J435, J438, J457, J458, J459, J460, J462,   \|  \| \| --- \| |
|  | CIHI-DAD | CCI | 3RF10VH, 3RF10VN, 3RF10XJ, 3RK10VH, 3RK10VN, 3RK10XJ, 3RZ12VA, 3RZ12VV, 2RM70BA, 2RM70CA, 2RM70DA, 2RM70LA |
| ***Prenatal visits*** | | | |
| CIHI-DAD | CIHI-DAD | ICD10 | Z34, Z35 |
|  |  | CCI | 5AB01, 5AB03 |
| Practitioner Claims | OHIP | Diagnostic code | 970 |
|  |  | Fee code | P003, P004, P005 |
| ***ODB indicator*** | | | |
| PIN | ODB | DIN | Defined as having at least 1 prescription in ODB for any drug in the 1 year prior to index date. |
| ***RAAS blockade*** | | | |
| PIN | ODB | DIN |  |
| ***Number of serum creatinine tests*** | | | |
| Laboratory | OLIS | OBSERVATIONCODE | 14682-9 |
| ***Renal proteinuria*** | | | |
| Laboratory | OLIS | OBSERVATIONCODE | 14959-1, 30000-4, 32294-1, XON10383-8, XON12394-3, 34366-5, 5804-0, 50561-0, 2887-8, 2888-6, 20454-5, 53525-2, 57735-3 |

Abbreviations: CIHI-DAD: Canadian Institutes for Health Information’s Discharge Abstract Database, CCI: Canadian Classification of Interventions, CORR: Canadian Organ Replacement Register; LDP: Longitudinal Demographic Profile; PIN: Pharmaceutical Information Network HYPER: Ontario Hypertension Dataset, IPDB: ICES Physician Database, NACRS: National Ambulatory Care Reporting System, ODB Ontario Drug Benefit, ODD: Ontario Diabetes Dataset, OHIP: Ontario Health Insurance Plan, OLIS: Ontario Laboratory Information System, RAAS: Renin-Angiotensin-Aldosterone System, RPDB: Registered Persons Database

**eTable 3: Assumed probability of CKD progression**

| Group | eGFR < 60 mL/min per 1.73 m^2^ | eGFR ≥ 60 mL/min per 1.73 m^2^ |
| --- | --- | --- |
| Pregnant group | CIF = 37.2%  Joint π = 0.002 | CIF = 3.0%  Joint π = 0.1998 |
| Non-pregnant group | CIF = 28.4%  Joint π = 0.0007 | CIF = 2.4%  Joint π = 0.7993 |

We expect the 5-year cumulative incidence of progression of CKD to be 2.4% in the non-pregnant group with an eGFR ≥ 60 mL/min per 1.73 m^2^. Using this group as a referent, we expect the following 5-year cumulative incidence of progression of kidney disease in each group to be 3.0% (corresponding to an RR of 1.25) in pregnant females with an eGFR ≥ 60 mL/min per 1.73 m^2^; 28.4% (corresponding to an RR of 11.8) in non-pregnant females with an eGFR < 60 mL/min per 1.73 m^2^; and 37.2% (corresponding to an RR of 15.5) in pregnant females with an eGFR < 60 mL/min per 1.73 m^2^. Our power calculation accounts for the differential probabilities in the 4 possible exposure combinations, assuming joint probabilities of the following: 79.93% non-pregnant females with eGFR ≥60, 19.98% pregnant females with eGFR ≥60, 0.07% non-pregnant females with eGFR <60 vs ≥60, and 0.02% pregnant females with eGFR <60.
